# Diagnosis and Management of Hypertension in Adolescents with Obesity

**DOI:** 10.1007/s12170-024-00740-x

**Published:** 2024-07-30

**Authors:** Shradha M. Chhabria, Jared LeBron, Sarah D. Ronis, Courtney E. Batt

**Affiliations:** 1 Department of Medicine, Case Western Reserve University School of Medicine, Cleveland, OH USA; 2 Department of Pediatrics, Case Western Reserve University School of Medicine, Cleveland, OH USA; 3 Division of Academic Pediatrics and Adolescent Medicine, UH Rainbow Babies & Children’s Hospital, 5805 Euclid Avenue, Cleveland, OH 44103 USA; 4 UH Rainbow Center for Child Health & Policy, Cleveland, OH USA; 5 University of South Florida Morsani College of Medicine, Tampa, FL USA

**Keywords:** Adolescent, Obesity, Hypertension, Elevated BMI, Elevated blood pressure

## Abstract

**Purpose of Review:**

Hypertension (HTN) and obesity are increasing in prevalence and severity in adolescents and have significant implications for long term morbidity and mortality. This review focuses on the diagnosis and management of HTN in adolescents with obesity with an emphasis on co-management of the two conditions.

**Recent Findings:**

Recent studies affirm the increasing prevalence of abnormal blood pressures and diagnoses of HTN associated with increased adiposity. Current guidelines recommend routine screening with proper technique for HTN in patients with obesity. Additionally, obesity and HTN related co-occurring medical conditions should be evaluated as there is frequently a bidirectional impact on risk and outcomes. Importantly, advances in adolescent obesity management have subsequently led to positive implications for the management of obesity-related comorbidities such as HTN. The co-management of obesity and HTN is an emerging strategy for treatment and prevention of additional morbidity and mortality as patients progress to adulthood.

**Summary:**

In adolescent patients with obesity, prompt recognition and appropriate diagnosis of HTN as well as related co-occurring conditions are necessary first steps in management. Co-management of obesity and HTN is likely to lead to improved outcomes. While lifestyle interventions serve as the foundation to this management, adjunctive and emerging therapies should be considered to adequately treat both conditions.

## Introduction

Affecting as many as 1 in 5 U.S. adolescents [54], obesity in this population has led to increased prevalence of obesity-related comorbidities, including hypertension (HTN), which affects up to 25% of adolescents with obesity, hereby referred to as “AWO,” and contributes to long term morbidity, mortality and health disparities as adolescents transition into adulthood [53]. Understanding the relationship between obesity and hypertension is essential for the appropriate care of adolescents and for optimizing the health of this population. Importantly, recent advances in obesity treatment, such as highly effective anti-obesity medications and bariatric surgery, have implications for the management of obesity-related comorbidities including HTN.

## Definition of Obesity and Hypertension in AWO

While many measures of adiposity exist, body mass index (BMI) or BMI percentile (Table 1) are most commonly used for screening. Recent studies indicate that as adiposity increases, so does mean systolic BP (SBP), mean diastolic BP (DBP), the prevalence of abnormally elevated BPs and the prevalence of HTN [68].

**Table 1:** Definitions of obesity in adolescents

	BMI percentile*	BMI*
Overweight	> 85 to < 95 percentile (%ile)	25 kg/m^2^ to < 30 kg/m^2^
Class 1 Obesity	> 95%ile to < 120% of the 95%ile	30 kg/m^2^ to < 35 kg/m^2^
Class 2 Obesity	> 120% of the 95%ile to < 140% of the 95%ile	35 kg/m^2^ to < 40 kg/m^2^
Class 3 Obesity	> 140% of the 95%ile	> 40 kg/m^2^

^*^When discrepancies exist, diagnosis based on whichever value is lower

For adolescents, abnormal blood pressure (BP) definitions rely on normative data for this population taking age, sex and height into account (Table 2). These definitions in the U.S. were most recently updated in 2017 based on evaluation of the existing literature and expert consensus [19]. BP percentiles are used for adolescents 10 to less than 13 years old. Exceptions exist when there is discordance in diagnosis between BP percentile and absolute BP (ex. BP percentile is normal, but absolute BP falls in the elevated blood pressure category) in which case, the lower value is used. In contrast, absolute BP values are used for adolescents 13 years and older to more closely reflect the adult guidelines [19].

**Table 2:** Definitions of hypertension in adolescents

	10 to < 13 years old*	> 13 years old
Normal	< 90 percentile (%ile)	< 120/ < 80 mmHg
Elevated Blood Pressure	> 90%ile to < 95%ile	120/ < 80 to 129/ < 80 mmHg
Stage 1 Hypertension	> 95%ile to < 95%ile + 12 mm Hg	130/80 to 139/89 mmHg
Stage 2 Hypertension	> 95%ile + 12 mmHg	> 140/90 mmHg

^*^or absolute BP values for adolescents ≥ 13 years old and adults, whichever is lower

Adapted from Flynn et al. [19]^4^

It is important to note that an increase in prevalence was observed in association with the updated definition of HTN in the 2017 guidelines [66], perhaps in part related to exclusion of data from children and adolescents with overweight or obesitywhen generating updated normative BP estimates [18]. A recent systematic review and meta-analysis found that the prevalence of abnormal SBP ranged from 0–16.5% in children and adolescents with heathy weight and increased to 5–45% of children and adolescents with class 1 obesity. Similarly, abnormal DBPs ranged from 4–9.7% in children and adolescents with healthy weight and increased to 9–29.4% in children and adolescents with class 1 obesity. Finally, a diagnosis of HTN was present in approximately 1% of children and adolescents with healthy weight and increased to 5% and 9% of children and adolescents with class 1 and class 2 obesity, respectively [68]. However, other large studies have estimated prevalence of HTN in AWO as high as 25% [19].

## Pathophysiology/Risk Factors

The pathophysiology of both obesity and HTN are complex. Obesity appears to be an independent risk factor for the development of HTN with several mechanisms by which obesity has been proposed to lead to HTN including *adiposopathy* and *fat mass disease*. Adiposopathy refers to dysfunction of adipocytes and adipose tissue, resulting in endocrine and immune dysregulation. Adiposopathy leads to activation of the sympathetic nervous system through insulin resistance, hyperinsulinemia, release of leptin and proopiomelanocortin and downregulation of adiponectin [26, 67, 78]. Additionally, inflammation associated with adiposopathy may affect endothelial function and vascular dilatory response [78]. Fat mass disease leads to renal compression and structural changes. These effects and the increased sympathetic outflow can in turn can lead to increase sodium retention and activation of the renin-angiotensin aldosterone system [64, 78].

Other risk factors that may contribute to the development of HTN in AWO include both modifiable and non-modifiable entities. Non-modifiable risk factors for HTN include sex, race/ethnicity and family history [19, 52]. Studies have demonstrated higher prevalence of elevated blood pressures in boys, adolescents (compared to younger children), those with parental history of early-onset HTN and Hispanic and non-Hispanic African American children [19, 52], though the interactions among race, obesity, and hypertension are not clear-cut. For example, in a study of 20,000 adolescents completing school-based blood pressure screening, African-American teens of healthy or overweight status had higher prevalence of hypertension compared to White or Hispanic peers, but the lowest prevalence of hypertension among those who had obesity [9]. Other modifiable risk factors include sub-optimal nutrition such as high sodium intake, lack of physical activity and substance use, particularly tobacco, and comorbidities including obstructive sleep apnea [19, 75]. Toxic stress resulting from adverse childhood events may also play a role in the development of HTN [2, 13, 15, 21, 71].

## Screening for and Diagnosis of Hypertension

Clinician awareness of pediatric HTN has increased over the past two decades, with screening frequency growing particularly among older patients and those with obesity [65]. Despite this, inconsistencies in screening rates across populations highlight a significant knowledge gap, with underdiagnosis in certain groups, such as African Americans [5, 6]. One study, for instance, revealed that up to 47% of pediatricians misclassified prehypertensive or hypertensive blood pressure readings as normal in at least one of six cases presented [3].

### Timing and Frequency of BP Screening

AAP guidelines advocate for annual BP screenings starting at age 3 and suggest monitoring BP at every healthcare visit for those with associated risk factors, such as obesity. Initial BP measurements can be taken using oscillometric devices, but elevations above the 90th percentile necessitate a confirmatory auscultatory reading, with the two results averaged. If elevated, further auscultatory readings are recommended, with follow-up timing based on the average BP level. Specifically, patients with elevated BP should have a repeat check in 6 months, those with Stage I hypertension should return in 1–2 weeks, and those with Stage II hypertension should start treatment and undergo diagnostic evaluations. Subsequent measurements should include both upper and lower extremity readings. Finally, Ambulatory Blood Pressure Monitoring (ABPM) should be used for confirmation across all elevated BP classes [19]^.^

However, concerns exist regarding the practicality of these guidelines, notably the variability in BP readings across visits, and the feasibility of the recommended frequent follow-ups. A study from Italy, for instance, highlighted a significant dropout rate of 37.9% among patients asked to return for follow-up BP checks within a 1–2 week period [14]. Moreover, despite ABPM being the gold standard, issues have been frequently reporting, including difficulties with access to devices due to cost and availability, and poor tolerability, underscoring challenges in adherence to these guidelines [27, 35].

### Proper BP Measurement

To measure BP, patients should be seated in a quiet room for 3–5 min before measurement, with no talking, a supported back, and feet uncrossed during measurement [19]. Both proper placement and cuff size are necessary for accurate readings. BP should be checked on the right arm unless the child has conditions that could lead to inaccurate readings from this position (e.g., atypical aortic arch anatomy). The arm should be at heart level, supported, and uncovered. The bladder length of the selected cuff should be 80–100% of the circumference of the arm, and the width should be at least 40% [57]. Therefore, offices should have multiple cuff sizes, this is particularly important for AWO given the increase in mid-upper arm circumference size associated with higher BMIs [57]. Prior research demonstrates that between 25–33% of children aged 9–11 with obesity will require an adult cuff. Some adolescents may require adult thigh cuffs applied to their arms for proper readings [56]. The use of wrist and forearm measurements is not recommended due to limited data on efficacy [19]. While approved oscillometric devices are permitted for initial measurement, auscultatory measurement is the most accurate mode of reading BP. Several studies have found overestimation and misclassification of BP with the use of oscillometric devices compared to auscultatory methods [1, 17].

### Diagnostic Evaluation

The majority of cases of HTN in AWO are primary HTN. Nevertheless, diagnostic evaluation is recommended to (1) rule out secondary causes of HTN, (2) identify target organ damage, and (3) assess for other cardiovascular risks. For all AWO with HTN, minimum workup should include urinalysis, chemistry panel including electrolytes, blood urea nitrogen and creatinine, and a lipid panel. If an abnormal urinalysis or renal function is noted, a renal ultrasound should be performed.

Based on patient history, evaluation for secondary causes may include: complete blood count, thyroid stimulating hormone, drug screen, and sleep study [10, 23, 47, 62]. Clinicians may also consider checking renin, endothelin, and protein induced by vitamin K absence (PIVKA-II) in adolescents with HTN in determining renal dysfunction, although this data is limited [33].

AWO with HTN requiring pharmacologic intervention should be evaluated with an echocardiogram to assess for cardiac target organ damage, including left ventricular hypertrophy (LVH) or reduced left ventricular ejection fraction (LVEF), given possible associations with long-term adverse outcomes [19]. This is particularly important as AWO have a 4.5 times higher odds of having LVH compared to adolescents with normal BMI, after adjusting for age, gender and BP [50]. Echocardiograms can be repeated in 6–12 month intervals in patients with persistent HTN despite treatment, concentric LVH or reduced LVEF for monitoring [19].

In addition, eye fundus examination for microcirculation damage may be helpful. Multiple studies have demonstrated worsened retinal arteriolar narrowing in AWO with higher blood pressures [40], and that lower central retinal arteriolar diameters can identify those who are at risk of developing higher SBP in the future [22, 46]. Assessment by fundus camera or optical coherence tomography could be considered given the ability to quantitatively analyze microvascular changes [45]. By contrast, routine screening for renal artery stenosis via renal imaging or microalbuminuria in AWO and HTN is not currently recommended due to insufficient evidence of benefit [19].

Finally, to identify additional cardiovascular risks in patients with BMI ≥ 95th percentile, hemoglobin A1C (A1C), aspartate aminotranferase and alanine aminotransferase (ALT), and fasting lipid panel should be performed [7, 32].

## Co-Morbid Conditions

Obesity and HTN are associated with several other diseases, including obstructive sleep apnea (OSA), dyslipidemia, Type 2 Diabetes Mellitus (T2DM), and metabolic dysfunction-associated steatotic liver disease (MASLD) all of which pose additional risks of sequelae and complications that may persist into adulthood. Therefore, we briefly review screening and primary interventions recommended for AWO with HTN. Notably, lifestyle management is the primary recommended intervention for each of these conditions. Additionally, adjunct and emerging therapies for the treatment of obesity are promising interventions for obesity-related comorbidities including HTN.

### OSA

OSA is deeply intertwined with both obesity and HTN. Obesity is a primary driver of adolescent OSA and HTN, and weight loss can help improve both conditions [10, 39]. Untreated OSA impairs sleep, and thereby increases energy consumption and weight gain. Therefore, treating OSA if indicated with adenotonsillectomy or CPAP can aid weight loss [28, 55]. Increased OSA severity is also associated with increased prevalence and severity of HTN [10]. Therefore, it is essential to simultaneously intervene on all three co-occurring diseases. AWO should be referred for polysomnography if they have any symptoms of disordered breathing, such as frequent snoring, gasps or labored breathing during sleep, or daytime sleepiness.

### Dyslipidemia

Among AWO, dyslipidemia typically presents as hypertriglyceridemia and low HDL, though elevated total cholesterol and LDL may also be seen [28]. The combination of obesity, HTN and dyslipidemia places adolescents at particularly high risk of future cardiovascular events including myocardial infarction and stroke [72].

### T2DM

Insulin resistance and hyperglycemia, commonly associated with both obesity and HTN, increases adolescents’ risk for prediabetes and T2DM [28]. T2DM is associated with micro- and macro-vascular damage in several organ systems, including cardiovascular disease, impaired renal function, retinopathy and neuropathy, many of which can be irreversible and progressive into adulthood [28].

### MASLD

MASLD (formerly non-alcoholic fatty liver disease, NAFLD) is a chronic liver disease mediated by insulin resistance and marked by hepatic steatosis, inflammation and fibrosis that can progress to steatohepatitis (MASH), increasing risk for cirrhosis and hepatic cancers [16, 28]. Compared to younger children, adolescents are more likely to develop worsening steatosis and less likely to experience resolution of MASH or regression of fibrosis [28]. Adults with MASLD have higher ambulatory blood pressures [73], and hypertensive adults with MASLD have increased risk of both cardiovascular and all-cause mortality [51].

## Management (Co-Management of Obesity and HTN)

The approach to co-managing HTN and obesity in adolescents must consider urgency, disease severity, co-occurring conditions and social determinants of health. Lifestyle modification is the foundational intervention, but multimodal approaches may be necessary to achieve disease control.

### Target BP Goals

The AAP recommends a treatment BP goal of < 130/80 mmHg. It is strongly recommended that obesity and HTN be treated concurrently to meet this goal, given that decreasing BMI has been shown to sustainably lower BP, with a 10 mm Hg decrease in SBP likely achievable with a 1.6 kg/m^2^ decrease in BMI [24, 28, 58, 60]**.**

### Lifestyle Modification

Lifestyle modification is the foundation of all management of obesity and HTN. Key steps include: dietary modifications, increased physical activity and improved sleep hygiene, with repeat assessment after 6 months [28]. Intensive health behavior and lifestyle treatments featuring at least 26 h of face-to-face, family-based contact time of multicomponent intervention are considered the standard-of-care, given improved efficacy in managing both diseases [28].

#### Dietary Recommendations

Given evidence for an apparent synergistic effect of obesity and high sodium intake on risk for elevated BP among adolescents, sodium restriction should be emphasized [79]. The Dietary Approaches to Stop Hypertension (DASH) diet focuses on sodium restriction to < 2300 mg/day, switching from high fat to low fat dairy products, reducing dietary sources of added sugars, consuming legumes, fish and lean meats, and increasing intake of fruits, vegetables and whole grains [28]. It has been associated with impressive BP reductions in adults, and when adapted for teens, has shown modest SBP improvements. Clinic-based behavioral nutrition intervention programs using the DASH diet were found to be more effective in reducing SBP among adolescents with HTN than routine care nutritional counseling [11, 12].

Reducing sugar-sweetened beverage consumption is also critical in preventing further weight gain [28]. Successful adherence to recommended changes may require adolescents to significantly decrease intake of fast food, prepackaged and processed foods, and to have access to produce. Clinicians should assess for and, whenever possible intervene on, food insecurity and other modifiable determinants influencing food selection.

#### Physical Activity

Pooled meta-analysis data of lifestyle interventions have shown that dietary interventions alone do not produce statistically significant improvement in obesity, with better efficacy using combined interventions including exercise and/or behavioral therapies [63]. Similarly, a recent systematic review and meta-analysis [30] found that physical activity interventions for children and adolescents were more effective at reducing blood pressure when paired with nutrition and behavior interventions. Adolescents are recommended to complete 60 min of moderate to vigorous physical activity daily. This can encompass a wide range of activities, including options that patients find fun, cooperative rather than competitive, and scalable as needed, particularly tailored to levels of fitness and any obesity-related comorbidities limiting activity [28]. For example, a school-based program combining lifestyle education with increased aerobic exercise through noncompetitive activity stations for 30 min thrice weekly demonstrated modest but significant reduction in SBP over just eight weeks [48]. Multiple additional studies have demonstrated improvements in elevated blood pressures with increased physical activity in children and adolescents [29, 41, 43].

### Adjunctive Therapies

Despite non-pharmacologic interventions, some adolescents will require adjunctive therapies to effectively manage both diseases, including anti-hypertensive medications, anti-obesity medications (AOMs) and/or bariatric surgery.

### Anti-Hypertensive Pharmacotherapy

The AAP recommends that anti-hypertensive medications be initiated for AWO who: 1) continue to have BP > 130/80 mmHg after six months of intervention, 2) have symptomatic HTN, or 3) have diabetes [28]. Others also recommend anti-hypertensive pharmacotherapy for secondary HTN or if evidence of hypertensive target organ damage is identified [25]. If anti-hypertensive medication is indicated, a single agent should be initiated at low-dose. First line agents include angiotensin-converting enzyme inhibitors (ACEis), angiotensin-receptor blockers (ARBs), long-acting calcium channel blockers (CCBs), and thiazide diuretics [28]. There is notably a dearth of RCT data to inform evidence-based decisions regarding relative efficacy or long-term outcomes among various anti-hypertensive agents in youth, particularly AWO. Nevertheless, short-term safety data has been reassuring, and all first line agents have shown some improvement in SBP and DBP, with the most data collected on ACEis and ARBs [59].

ACEis and ARBs are particularly favored as first-line agents in concomitant obesity and HTN, given metabolically favorable profiles and the possibility of renal protection against obesity-related hyperfiltration and/or T2DM [4, 19, 28]. Pregnancy is a contraindication to these agents due to risk of fetal toxicity, and contraceptive counseling and options must be provided to adolescent patients prior to initiation of therapy. Angioedema is a contraindication to ACEis but not ARBs.

Similar to ACEis and ARBs, many CCBs can be dosed daily and do not have known metabolic side effects, thus are generally considered favorable for patients with obesity. CCBs can commonly cause peripheral edema and flushing, with hypersensitivity to CCBs the only contraindication. Thiazide diuretics can be useful agents in lowering BP, however have been found in adult studies to be associated with new onset DM and elevated fasting glucose, thus raising some concern about use in patients with obesity and HTN [36]. Thiazides are contraindicated in anuria and can also cause hypokalemia, cardiac dysrhythmias, pancreatitis and cholestatic jaundice [19].

If BP is not well controlled with a single agent, a second agent can be added and titrated. Other anti-hypertensive agents such as alpha-blockers, potassium-sparing diuretics and direct vasodilators are typically reserved for patients not responsive to two or more of the preferred agents [19]. Beta-blockers are known to have unfavorable side effects including increased weight gain and dyslipidemia, and should be avoided in patients with obesity.

If lifestyle changes and weight reduction are successful in significantly reducing BP, anti-hypertensive agents may be reduced or discontinued with close monitoring.

### Anti-Obesity Pharmacotherapy

AWO and HTN may also benefit significantly from the use of pharmacologic and/or surgical therapies to augment weight loss. Current guidelines support the use of anti-obesity medications in adolescent patients with a BMI ≥ 95%ile [28, 69, 70]. It is important to note that some AOMs have known teratogenic effects or are otherwise not recommended in patients who are currently or may become pregnant while on treatment. Therefore, it is essential for adolescents considering AOMs to receive contraceptive counseling with options available. Furthermore, there is significant risk of weight regain associated with discontinuation of AOMs, thus long-term use may be required for effective weight management.

There are currently four anti-obesity medications approved for long-term use by AWO by the US Food and Drug Administration (FDA): orlistat (Xenical, Alli), combined phentermine-topiramate (Qsymia), daily liraglutide (Saxenda) and weekly semaglutide (Wegovy). Setmelanotide (Imcivree) is also FDA approved for use in specific forms of monogenic obesity or in Bardet-Biedl syndrome in patients 6 years of age and older.

Orilstat is a gastrointestinal lipase inhibitor that reduces systemic fat absorption, found to produce modest mean BMI change of 0.83 kg/m^2^ with no significant on metabolic indicators compared to placebo in adolescents. However, there is a 50% increase in gastrointestinal side effects including oily spotting of underwear, as well as increase in fecal incontinence and flatus with discharge, rendering this mild to modestly effective medication intolerable for many [74].

Qysmia is a combined formulation of phentermine and topiramate that works centrally by mediating norepinephrine, gamma-aminobutyric acid and glutamate to produce appetite suppression. It can produce an approximately 8–10% reduction in BMI among adolescents over 1 year [38]. It is important to note that the 2022 study by Kelly et al. establishing safety and efficacy of Qsymia in adolescents excluded those with baseline elevated BP from the study [38]. However, in contrast to prior warnings about possible increases in BP with phentermine monotherapy due to expected amphetamine activity, hypertension is not a FDA-reported contraindication to use of Qsymia. Rather, it notes that the effects of Qsymia on heart disease or related mortality have not been established, and recommends close BP monitoring given risk of hypotension if Qsymia is used in patients already on anti-hypertensive medications [42, 61]. This is supported by RCT data showing net decreases in mean SBP and DBP alongside weight loss with use of Qsymia [31, 34, 38, 42].

Liraglutide and semaglutide are among several injectable glucagon-like peptide 1 receptor agonist (GLP-1 RA) medications that can produce very effective weight loss by targeting both central and peripheral organ targets. GLP-1 is naturally secreted by the small and large intestines, with primary effects exerted by reducing appetite stimulation in the hypothalamus, slowing gastric emptying, increasing post-prandial insulin release and decreasing glucagon secretion. There is also evidence of positive metabolic effects on glucose regulation, lipid metabolism and cardioprotection [8]. GI side effects including nausea and vomiting are common with GLP-1 RAs, particularly at initiation with improvement over time [37, 76]. Weight regain after treatment discontinuation is also marked, with data from adult extension trials of once-weekly semaglutide demonstrating mean regain of two-thirds of weight lost in one year following cessation of treatment [77].

Daily liraglutide (Saxenda) was approved by the FDA for use by adolescents in 2020, after Kelly et al. demonstrated a mean reduction of -4.6% of BMI and 5% of body weight (4.5 kg on average), with 43% of participants achieving reduction in BMI of at least 5% in an RCT over 56 weeks of treatment. Patients with uncontrolled treated or untreated HTN above 99th percentile for age and gender were excluded from the trial, and changes in BP between treatment and placebo arms were not significant [37]. In 2022, the FDA approved Wegovy, once-weekly semaglutide for adolescents, after Weghuber et al. found a ~ 16% mean decrease in BMI compared to placebo over 68 weeks, with an impressive 73% of participants in trial arm experiencing weight loss of at least 5%. While 13% of participants had HTN (treated or untreated) at baseline, there were no differences in BP noted between treatment and placebo groups [76]. However, the FDA recently approved use of Wegovy for risk reduction of cardiovascular death, heart attack and stroke among adults with overweight or obesity, after a multicenter study found a 20% reduction in these outcomes among those under treatment [44]. Though not yet evaluated in adolescents, this is a promising glimpse into possible long-term cardiovascular benefit for AWO and HTN on GLP-1 RA therapy.

Several other medications are used off-label from FDA approval for weight loss in adolescents. Metformin is a biguanide anti-diabetic medication that has shown modest efficacy (BMI reduction ~ 1 kg/m^2^) for weight loss compared to lifestyle therapy alone [69]. Phentermine is FDA approved as monotherapy for short term use (up to 12 weeks duration) among adolescents 17 years and older, but is contraindicated in uncontrolled hypertension and pregnancy. It has been shown to produce ~ 4% mean BMI reduction at six months and is used off-label for durations longer than FDA approval. However, it is a Class IV controlled substance, and state bylaws and statues should be followed [69]. Topiramate monotherapy has been found to produce 4–6% reduction in BMI in adolescents with severe obesity. It is also teratogenic and may decrease efficacy of oral contraception [20, 69].

Finally, at the time of writing, there are several other AOMs under study for the treatment of obesity in both the adult and adolescent populations including tirzepatide, a GLP1/ glucose-dependent insulinotropic polypeptide (GIP) co-agonist which is approved in adult patients and currently undergoing trials in adolescents as well as novel amylin agonists and peptide YY agonists [49]. While beyond the scope of this review, the development of additional medications for the treatment of obesity may add to the providers’ toolbox for treatment of obesity and HTN in the future.

### Bariatric Surgery

Lastly, bariatric surgery including sleeve gastrectomy or Roux-en-Y gastric bypass should be considered in adolescents with Class 2 obesity and a related comorbidity including HTN or Class 3 obesity [28]. Adolescents have been found to have durable reduction and BMI and higher probability of remission of HTN and T2DM compared to adults undergoing bariatric surgery. Eligible and interested adolescents should be referred to a comprehensive metabolic and bariatric surgery center [28].

## Conclusions and Summary

While the prevalence of obesity and HTN is increasing, so, too, are recognition and the tools for management of these chronic medical conditions in the adolescent population. The initial steps of identification and diagnosis are key for the successful treatment of patients with these co-occurring conditions. Recent and emerging advances in therapies for obesity provide promising avenues for future treatment of adolescent patients with obesity and HTN.

## Data Availability

No datasets were generated or analysed during the current study.
